# Uncovering the Early Events Associated with Oligomeric Aβ-Induced Src Activation

**DOI:** 10.3390/antiox12091770

**Published:** 2023-09-16

**Authors:** Sandra I. Mota, Lígia Fão, Patrícia Coelho, A. Cristina Rego

**Affiliations:** 1 CNC-UC-Center for Neuroscience and Cell Biology, University of Coimbra, 3004-504 Coimbra, Portugal; sandra.mota@cnc.uc.pt (S.I.M.); ligia.fao@cnc.uc.pt (L.F.); prcoelho@cnc.uc.pt (P.C.); 2 CIBB-Center for Innovative Biomedicine and Biotechnology, University of Coimbra, 3004-504 Coimbra, Portugal; 3 IIIUC-Institute of Interdisciplinary Research, University of Coimbra, 3030-789 Coimbra, Portugal; 4 Institute of Biochemistry, Faculty of Medicine, University of Coimbra, 3000-354 Coimbra, Portugal

**Keywords:** Alzheimer’s disease, Src tyrosine kinase, NMDA receptor, mitochondrial dysfunction, mitochondrial morphology

## Abstract

Soluble Aβ_1–42_ oligomers (AβO) are formed in the early stages of Alzheimer’s disease (AD) and were previously shown to trigger enhanced Ca^2+^ levels and mitochondrial dysfunction via the activation of *N*-methyl-D-aspartate receptors (NMDAR). Src kinase is a ubiquitous redox-sensitive non-receptor tyrosine kinase involved in the regulation of several cellular processes, which was demonstrated to have a reciprocal interaction towards NMDAR activation. However, little is known about the early-stage mechanisms associated with AβO-induced neurodysfunction involving Src. Thus, in this work, we analysed the influence of brief exposure to oligomeric Aβ_1–42_ on Src activation and related mechanisms involving mitochondria and redox changes in mature primary rat hippocampal neurons. Data show that brief exposure to AβO induce H_2_O_2_-dependent Src activation involving different cellular events, including NMDAR activation and mediated intracellular Ca^2+^ rise, enhanced cytosolic and subsequent mitochondrial H_2_O_2_ levels, accompanied by mild mitochondrial fragmentation. Interestingly, these effects were prevented by Src inhibition, suggesting a feedforward modulation. The current study supports a relevant role for Src kinase activation in promoting the loss of postsynaptic glutamatergic synapse homeostasis involving cytosolic and mitochondrial ROS generation after brief exposure to AβO. Therefore, restoring Src activity can constitute a protective strategy for mitochondria and related hippocampal glutamatergic synapses.

## 1. Introduction

Alzheimer’s disease (AD) stands as the most prevalent neurodegenerative condition and a primary cause of dementia among the elderly population. It is characterised by the accumulation of extracellular senile plaques formed by amyloid-beta peptide (Aβ) and intracellular neurofibrillary tangles composed of hyperphosphorylated Tau [1]. AD is thought to stem from numerous early pathological processes that emerge decades before the onset of symptoms, ultimately leading to the loss of synaptic plasticity and cellular demise [2]. Synaptic function largely depends on the actin cytoskeleton and high levels of ATP [3]. In this perspective, mitochondria are the neuron’s most efficient way of producing energy, also participating in Ca^2+^ storage and signaling, reactive oxygen species (ROS) production and control of apoptotic pathways [4,5]. Mitochondrial dysfunction has emerged as a key early event in AD pathogenesis triggered by Aβ oligomers (AβO) through both direct and indirect pathways. Indeed, soluble AβO_1–42_ were shown to trigger mitochondrial Ca^2+^ rise and depolarization of the mitochondrial membrane via activation of *N*-methyl-D-aspartate receptors (NMDAR), contributing to neuronal dysfunction [6]. Furthermore, within mitochondria, Aβ interacts with various proteins, such as the voltage-dependent anion-selective channel 1 (VDAC1) [7], dynamin-related protein 1 (Drp1) [8] or cytochrome C oxidase subunit 1 [9]. These interactions disrupt mitochondrial dynamics and function, ultimately leading to synaptic and neuronal damage and, consequently, cognitive decline in AD patients.

Src family kinase (SFK) is a family of non-receptor tyrosine kinases involved in several cellular processes, namely cell differentiation, signal transduction and cellular metabolism [10,11,12], as well as synaptic plasticity through the modulation of NMDARs [13]. SFKs are redox-sensitive and can be directly or indirectly activated by hydrogen peroxide (H_2_O_2_) [14]. Src is a ubiquitously expressed member of SFK [15,16] involved in several cellular processes and is activated by ROS [14]. Importantly, Src regulates neuronal plasticity and behavior through the phosphorylation of the GluN2B subunit of NMDARs at Tyr1472, increasing NMDAR’s targeting of the synaptic membrane [17]. If, on the one hand, Src induces NMDARs activity, on the other hand, prolonged NMDAR activation further promotes Src activity. Importantly, Src was also identified in the intermembrane space of highly purified rat brain mitochondria [18]. In mitochondria, Src can modulate brain mitochondrial respiration [19], linking ROS signaling with mitochondrial function.

In previous studies, we demonstrated that AβO directly interact with NMDAR subunits [20] evoking a transient increase in intracellular Ca^2+^ (Ca^2+^_i_) [21]. Sustained activation of NMDARs and AβO massively increase Ca^2+^_i_, which is rapidly taken up by mitochondria [6], increasing ROS generation [22]. Importantly, we previously observed a decrease in Src activation/phosphorylation in the hippocampus of 3-month-old 3xTg-AD male mice but enhanced Src activation in the hippocampus of 15-month-old 3xTg-AD female mice, both occurring concomitantly with similar changes in phosphorylation of the GluN2B subunit at Tyr1472 [23]. Thus, evidence suggests that Aβ-induced synaptic dysfunction is dependent on NMDARs and occurs via aberrant redox events, potentially modifying redox-sensitive proteins, such as Src. However, little is known of whether early AβO-induced hippocampal neurodysfunction and dendritic impoverishment are linked to altered Src activation. Thus, we evaluated the influence of brief exposure to AβO on the activation of Src kinase and further determined the role of NMDAR activation, redox changes and mitochondria on Src changes in mature primary rat hippocampal neurons. Our results show that a short incubation with AβO evoke ROS-dependent Src activation involving NMDARs and resulting increase in Ca^2+^_i_, as well as unbalanced cytosolic and mitochondrial H_2_O_2_ levels and mild mitochondrial fragmentation. Our study supports an important role for Src kinase in early AβO exposure as a process contributing to continuous glutamatergic postsynaptic dysfunction and mitochondrial changes in AD.

## 2. Materials and Methods

### 2.1. Materials

Neurobasal medium, gentamicin, B27 supplement, fetal bovine serum (FBS) and all antibiotics were purchased from GIBCO (Paisley, UK). The synthetic Aβ_1–42_ and Aβ_42–1_ peptide was obtained from Bachem (Bubendorf, Switzerland). Protease cocktail inhibitors, Fura-2/AM, Amplex^®^ Red were purchased from Invitrogen/Molecular Probes (Life Technologies Corporation, Carlsbad, CA, USA). The compound MK-801 [(+)-5-methyl-10,11-dihydro-5H-dibenzo[a,d] cyclohepten-5,10-imine maleate)] was obtained from Calbiochem (Merck Millipore, Darmstadt, Germany). Bradford protein assay was purchased from BioRad Laboratories, Inc. (Munich, Germany). TMRM^+^ probe (tetramethylrhodamine methyl ester), acrylamide, methanol, acetic acid and secondary antibodies used in Western blotting were purchased from Thermo Fisher Scientific (Rockford, IL, USA). BSA used in Western blotting was purchased from Santa Cruz Biotechnology (Santa Cruz Biotechnology, Inc., Dallas, TX, USA). ECF substrate and Western Blot PVDF membrane were purchased from GE Healthcare (Chicago, IL, USA). Trypsin, trypsin inhibitor, fatty acid free bovine serum albumin (BSA), Modified Eagle’s Medium (MEM) culture medium (M0268), Dulbecco′s Modified Eagle′s Medium (DMEM) culture medium (D5030), SU6656, L-Glutathione ethyl ester (GSH-EE), N-acetyl-L-cysteine (NAC), hydrogen peroxide (H_2_O_2_), 5-fluoro-2′-deoxyuridine (5-FDU), horseradish peroxidase, MitoPY1, anti-β-actin antibody (A5316) and other analytical grade reagents were purchased from Sigma Chemical and Co. (St. Louis, MO, USA). Antibodies against Nrf2 (ab31163-500) and Phospho-Nrf2 (S40) (ab76026) were from Abcam (Cambridge, UK). Antibodies against Src (#2110) and Phospho-Src (Tyr 416) (#6943) were from Cell Signaling Technology (Danvers, MA, USA).

### 2.2. Primary Hippocampal Culture

Primary hippocampal neuron cultures were prepared, as described previously, in the presence of 5-fluoro-2′-deoxyuridine [24]. All animal experiments were carried out in accordance with the guidelines of the Institutional Animal Care and Use of Committee and the European Community directive (2010/63/EU) and protocols approved by the Faculty of Medicine, University of Coimbra (ref: ORBEA_211_2018) and the Direção Geral de Alimentação e Veterinária (DGAV, ref: 0421/000/000/2019). All efforts were made to minimise animal suffering and to reduce the number of animals used.

### 2.3. AβO Preparation

AβO preparation and Aβ_42–1_ oligomeric preparation were obtained, as previously described in [17]. Briefly, synthetic Aβ peptide was dissolved in cold 1,1,1,3,3,3-hex- afluoro-2-propanol (HFIP) to a final concentration of 1 mM and aliquoted. The peptide-HFIP solutions were incubated at room temperature for 60 min, followed by 5–10 min incubation on ice. HFIP was first evaporated overnight in the hood at room temperature, and then the remaining was removed in a Speed Vac (Ilshin Lab. Co. Ltd., Ede, The Netherlands). Dried HFIP film was stored at −80 °C and, when necessary, resuspended to make a 5 mM solution in anhydrous dimethyl sulfoxide and then dissolved in phenol red-free Ham’s F-12 medium without glutamine to a final concentration of 100 μM and incubated overnight at 4 °C. The preparation was centrifuged at 14,000× *g* for 10 min at 4 °C to remove insoluble aggregates, and the supernatant containing soluble oligomers and monomers was transferred to prelubricated clean tubes (Costar) and stored at −20 °C. Protein content was determined using the BioRad protein assay and quantified using a microplate reader Spectra Max Plus 384 (Molecular Devices, San Jose, CA, USA). The presence of different assembly peptide forms (monomers, oligomers and/or fibrils) in the preparation was evaluated by 4–16% nondenaturing Tris–Tricine polyacrylamide gel electrophoresis and further staining with a solution of 0.5% Coomassie in 45% methanol and 10% acetic acid. Typically, our preparations showed only small oligomers (16 to 24 kDa) and no monomers or fibrils (data not shown). 

### 2.4. Experimental Conditions

Mature primary hippocampal neurons (17–18 DIV) were exposed to 1 µM of soluble Aβ_1–42_ oligomers (AβO) for 5, 10 or 30 min or acutely. When indicated, the reverse peptide Aβ_42–1_ was used to confirm the specific effect of AβO. To evaluate the role of oxidative stress in AβO-induced effects, cells were pre-exposed to antioxidants GSH (0.1 mM) and Mitotempo (MT; 1 µM), a mitochondrial antioxidant, for 24 h and N-acetyl-L-cysteine (NAC; 1 mM), a precursor of GSH, for 1 h. The effect of Src inhibition was also assessed by using SU6656 (5 µM) after 1 h pretreatment [18], as well as the effect of NMDARs inhibition by using MK-801 (10 μM; 10 min pretreatment). All incubations were performed in a conditioned culture medium. For live experiments, cells were maintained in Mg^2+^-free Na^+^ medium (containing 140 mM NaCl, 5 mM KCl, 1 mM CaCl_2_, 10 mM Glucose, 10 mM Hepes, pH 7.4/NaOH) supplemented with glycine (20 µM) and serine (30 µM). 

### 2.5. Protein Extraction and Western Blotting

Total extract protein was obtained after washing cells 3 times in ice-cold PBS and then scraping them in RIPA extraction buffer (containing 150 mM NaCl, 50 mM Tris HCl, 5 mM EGTA, 1% Triton X-100, 0.1% SDS, 0.5% deoxycholate, pH 7.5) supplemented with 100 nM okadaic acid, 1 mM PMSF, 25 mM NaF, 1 mM Na_3_VO_4_, 1 mM DTT and 1 μg/mL protease inhibitor cocktail (chymostatin, pepstatin A, leupeptin and antipain). Homogenates were then lysed in an ultrasonic bath (UCS 300—THD; at heater power 200 W and frequency 45 kHz) for 10 sec and centrifuged for 10 min at 20,800× *g* (4 °C) to remove cell debris, and the supernatant was collected. Protein content was determined using the Bradford protein assay. Equivalent amounts of protein samples (20 μg) were denaturated at 95 °C for 5 min with 6x concentrated loading buffer (containing 300 mM Tris-HCl pH 6.8, 12% SDS, 30% glycerol, 600 mM DTT, 0.06% bromophenol blue), and separated by 8–12% SDS-PAGE gel electrophoresis and electroblotted onto polyvinylidene difluoride (PVDF) membranes. Membranes were further blocked 1h at room temperature with 5% (*w*/*v*) BSA in Tris Buffered Saline (containing Tris-HCl 25 mM pH 7.6 and NaCl 150 mM) with 0.1% Tween-20 (TBS-T) and then incubated overnight at 4 °C with primary antibodies: β Actin (1:5000), Nrf2 (1:500), Phospho-Nrf2 (S40) (1:500), Src (1:1000); Phospho-Src (Tyr 416) (1:1000). Antimouse or antirabbit IgG secondary antibody conjugated to the alkaline phosphatase (1:10,000) prepared in 1% (*w*/*v*) BSA in TBS-T was used for 1 h at room temperature. Immunoreactive bands were visualised by alkaline phosphatase activity after incubation with ECF reagent and visualised by using a BioRad ChemiDoc Touch Imaging System (BioRad, Hercules, CA, USA) and quantified using Image Lab analysis software (BioRad).

### 2.6. Intracellular Calcium Levels

Primary hippocampal neurons were incubated with a 10 μM Fura-2/AM fluorescent probe for 40 min at 37 °C in a conditioned medium before pre-incubation ending. After a washing step with Na^+^ medium (containing 140 mM NaCl, 5 mM KCl, 1 mM CaCl_2_, 1 mM MgCl_2_, 10 mM Glucose, 10 mM Hepes, pH 7.4/NaOH), Fura-2 fluorescence was monitored in Mg^2+^-free Na^+^ medium plus serine/glycine using a Spectrofluorometer Gemini EM (Molecular Devices, San Jose, CA, USA) microplate reader at a 340/380 nm excitation and 510 nm emission wavelengths. Fura-2 fluorescence was recorded for 2 min (basal values) and a further 5 min after stimuli with AβO. Fluorescence values (ratio 340/380) were normalised to the baseline.

### 2.7. Measurement of Cellular H_2_O_2_ Levels

H_2_O_2_ released by primary hippocampal neurons was determined using the extracellular Amplex^®^ Red assay through the monitoring of resorufin fluorescence (excitation 550 nm; emission 580 nm), the stoichiometry product of H_2_O_2_ reacting with Amplex^®^ Red reagent (10-acetyl-3,7-dihydroxyphenoxazine). After a washing step with Na^+^ medium, H_2_O_2_ levels were measured in Mg^2+^-free Na^+^ medium plus serine/glycine supplemented with 10 µM Amplex^®^ Red plus 0.5 units/mL of horseradish peroxidase for 3 min (basal) and for 30 min after stimuli using a microplate reader Spectrofluorometer Gemini EM (Molecular Devices, San Jose, CA, USA).

### 2.8. Evaluation of Mitochondrial H_2_O_2_ Levels

MitoPY1 was used to measure mitochondrial-derived H_2_O_2_ in primary hippocampal neurons. Cells were incubated with 10 µM MitoPY1 in Na^+^ medium at 37 °C for 30 min. Then, cells were washed to remove the not-internalised probe, and changes in mitochondrial H_2_O_2_ levels were analysed in Mg^2+^-free Na^+^ medium plus serine/glycine using confocal images obtained using a 20× objective with NA = 0.8 on a Carl Zeiss Axio Observed Z1 inverted confocal microscope using the CSU-X1M spinning disc technology (Zeiss, Jena, Germany). Basal mitochondrial H_2_O_2_ levels were recorded for 15 min basal followed by 30 min after stimuli (one frame every minute). Fluorescence intensity was quantified using FIJI software (version 2.1.0/1.51w).

### 2.9. Mitochondrial Membrane Potential Assessment

Primary hippocampal neurons were incubated in a conditioned culture medium with the mitochondrial membrane potential (mmp)-sensitive probe (TMRM^+^ at 300 nM, under quench mode) for 30 min in the incubator (37 °C, 5% CO_2_) before the end of AβO incubation. After a washing step with Na^+^ medium, mmp was measured in cell population in Mg^2+^-free Na^+^ medium plus serine/glycine, plus TMRM^+^, using a microplate reader Spectrofluorometer Gemini EM (Molecular Devices, San Jose, CA, USA) (540 nm excitation, 590 nm emission). Changes in mmp were assessed by the analysis of TMRM^+^ fluorescence dequenching after complete mitochondrial depolarization (mmp collapse) achieved by adding a protonophore [2 μM carbonyl cyanide-4-(trifluoromethoxy)phenylhydrazone (FCCP)] plus oligomycin (2 μg/mL) to prevent ATP synthase reversal. 

### 2.10. Cell Transfection

Primary hippocampal neurons were cotransfected with plasmids codifying for MitoDsRed (to label mitochondria) plus GFP (to fill in the cell) when still immature (at 8 DIV) using calcium phosphate coprecipitation protocol as described in [24].

### 2.11. Evaluation of Mitochondrial Morphology

Mitochondrial morphology was assessed as in [24]. Briefly, hippocampal neurons, cotransfected with MitoDsRed plus GFP, were washed and incubated in Na^+^ medium plus serine/glycine at 37 °C for mitochondrial movement studies. An image of neuronal projections (MitoDSRed plus GFP) was acquired using a 63× objective with NA = 1.4 on a Carl Zeiss Axio Observed Z1 inverted confocal microscope using the CSU-X1M spinning disc technology (Zeiss, Jena, Germany) after 10 min incubation with AβO in the absence or presence of SU6656 or MK-801. To assess mitochondrial morphology, the macros AutoROI and MitoProtAnalyser for FIJI were applied in the last image acquired to assess the following parameters per mitochondria: aspect ratio (major axis/minor axis) and circularity (4π × area/perimeter^2^), which reflect length and degree of fragmentation, respectively. 

### 2.12. Statistical Analyses

Data were expressed as the mean ± SEM of the number of experiments or elements (neuritis or mitochondria) indicated in the figure legends. The normal distribution of each population was analysed, and all experimental groups were considered nonparametric. Thus, comparisons among multiple groups (relative to control or AβO treatment) were performed by nonparametric one-way analysis of variance (ANOVA) followed by the Kruskal-Wallis Multiple Comparison post hoc test. The Mann-Whitney U-test was also performed for comparison between the two populations, as described in figure legends. Significance was defined as *p* < 0.05.

## 3. Results

### 3.1. AβO Induce Src Kinase Activation Involving NMDARs Activation and Enhanced H_2_O_2_ Levels

**Figure 1:**
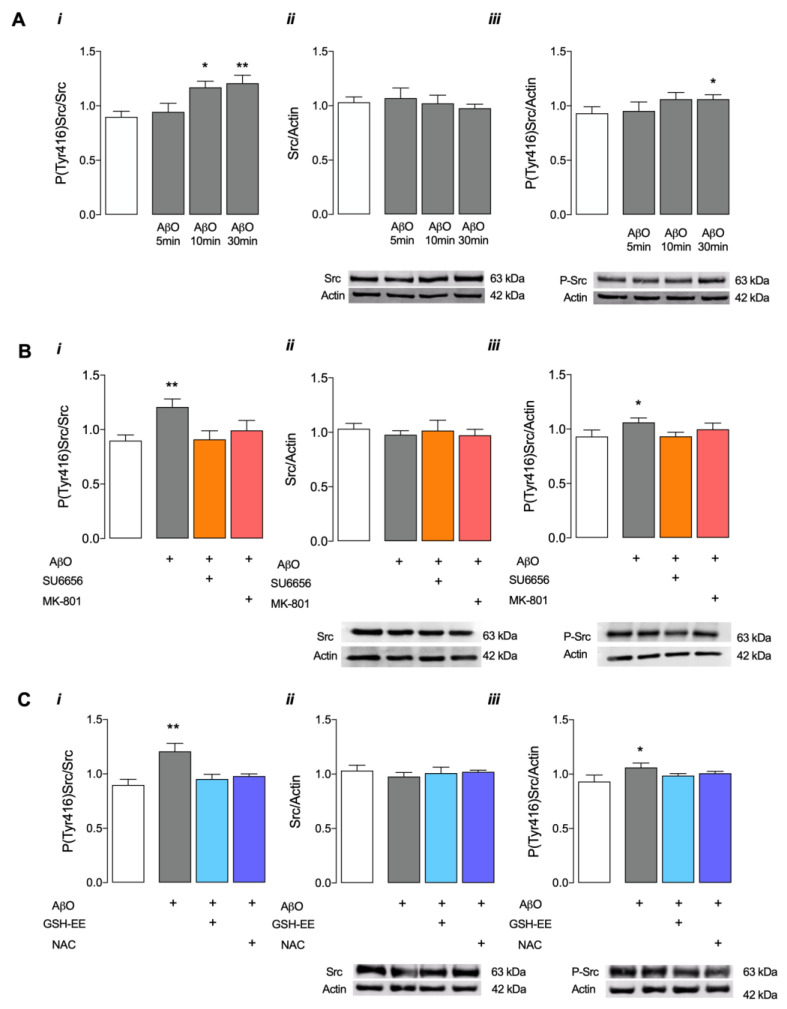
Src total and phosphorylated protein levels in mature hippocampal neurons after exposure to AβO. Hippocampal mature neurons (17 DIV) were incubated with 1 μM AβO for 5, 10 and 30 min in (**A**), and the levels of P(Tyr416)Src/Src (**A*i****,***B*i****,***C*i***), Src/actin (**A*ii****,***B*ii****,***C*ii***) and P(Tyr416)Src/actin (**A*iii****,***B*iii****,***C*iii***) were evaluated using Western blotting. The effects of SU6656 (5 μM) and MK-801 (10 μM) in (**B**), as well as NAC (1 mM) and GSH-EE (0.1 mM) in (**C**), were evaluated in cells exposed to AβO (1 μM), for 30 min. Data are expressed in arbitrary units as the mean ± SEM of n = 3 to 10 experiments. Statistical analysis: * *p* < 0.05 and ** *p* < 0.01 vs. control (Kruskal-Wallis followed by Dunn’s post hoc test).

The effect of brief exposure to AβO (1 μM) on the activation of the redox sensor protein, the nonreceptor Src tyrosine kinase family (Src), was first investigated in mature primary hippocampal neurons. We analysed the levels of total and phosphorylated Src at residue Tyr416, which reflects its activation [25], in hippocampal neurons treated with AβO for up to 30 min in the absence or presence of pharmacological compounds (Figure 1). The concentration of AβO was selected based on the levels of soluble Aβ_1–42_ determined in pyramidal neurons derived from the AD patient’s hippocampus [~3 μM [26]] and on previous results obtained in the group in both hippocampal [24] and cortical primary neurons [6,21].

Results shown in Figure 1A and Figure S3 evidence that exposure for 10 and 30 min to AβO causes an increase in the levels of phosphorylated Src (iii) and P(Tyr416)Src/Src ratio (i) while maintaining the levels of total Src (ii) when compared to the control condition (i.e., nontreated cells). Phosphorylation of Src at residue Tyr 416 reflects its activation [16], largely suggesting that a short exposure to AβO induces Src activation.

To confirm that under these conditions Src activation could be modulated by NMDAR activation and ROS levels, we pre-incubated cells with MK-801 (Figure 1B) or with antioxidants (Figure 1C and Figure S5) and determined the influence of AβO incubated for 30 min. Pretreatment with MK-801, a noncompetitive NMDAR inhibitor, prevented the phosphorylation/activation of Src induced by AβO (Figure 1B and Figure S4), suggesting that activation of NMDARs is required for AβO-induced Src activation. Of note, pre-incubation with SU6656, an inhibitor of the SFK, similarly inhibited AβO-evoked Src activation (Figure 1B and Figure S4). Furthermore, AβO-induced increase in P(Tyr416)Src levels was largely prevented in cells pretreated with both antioxidants GSH-EE (a cell-permeable derivative of reduced glutathione, GSH) and NAC (a precursor of GSH synthesis by providing Cys), suggesting that the activation of Src is also modulated by oxidative events. Results obtained in Figure 1 evidence that short exposure of mature hippocampal neurons to AβO induces the phosphorylation/activation of Src kinase in a process mediated by both NMDARs and redox changes.

**Figure 2:**
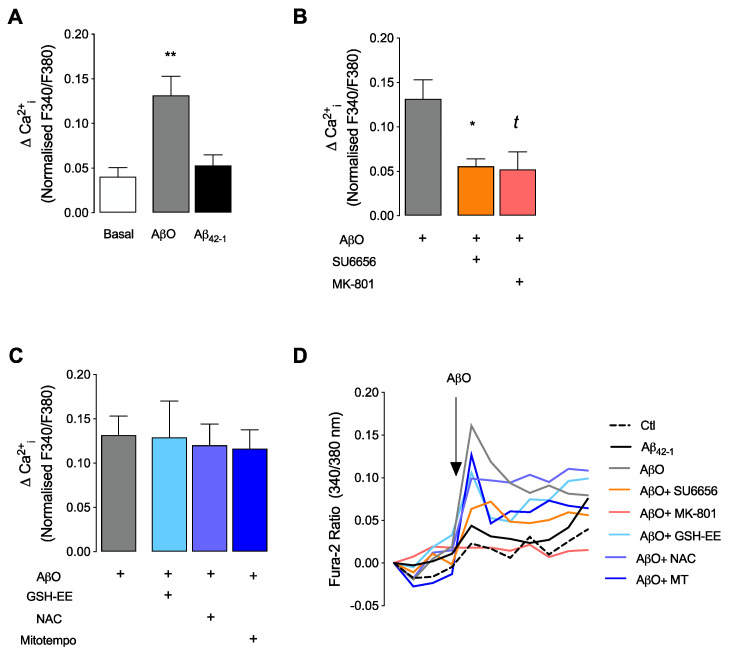
Intracellular Ca^2+^ levels after acute treatment with AβO in mature hippocampal neurons. Basal Ca^2+^_i_ levels were recorded for 2 min in mature hippocampal neurons (17 DIV), and the effect of AβO (1 μM) was recorded for a further 5 min. The effect of AβO was calculated by analysing the Fura-2 fluorescence ratio at 340/380 nm. The effect of the reverse peptide AβO_42–1_ (1 μM) was evaluated in (**A**). The effects of SU6656 (5 μM) and MK-801 (10 μM) were analysed in (**B**). The influence of antioxidants GSH-EE (0.1 mM), NAC (1 mM) and Mitotempo (MT; 1 μM) was evaluated in (**C**). Results were plotted as the difference between the last and the first values achieved before and after the addition of AβO. Graphic (**D**) is the representative line chart (normalised to baseline). Data are expressed as the mean ± SEM of n = 3 to 10 experiments, run in triplicates. Statistical analysis: * *p* < 0.05 and ** *p* < 0.01 vs. control (Kruskal-Wallis followed by Dunn’s post hoc test), *^t^ p* < 0.05 (Mann-Whitney).

To further support the involvement of NMDARs on AβO-induced Src activation, we measured Ca^2+^_i_ levels (Figure 2) following immediate exposure to AβO in the absence or presence of MK-801, SU6656 and antioxidants. AβO (1 μM) evoked an immediate rise in Ca^2+^_i_ in mature hippocampal neurons (Figure 2A,D). Of relevance, this effect was specific to the Aβ_1–42_ oligomeric form since the reverse peptide Aβ_42–1_ did not induce a significant change in Ca^2+^_i_ (Figure 2A,D). Figure 2B shows that prior inhibition of NMDARs using MK-801 completely prevented the entry of Ca^2+^ through the receptor. Similarly, prior inhibition of Src kinases with SU6656 also prevented the rise in Ca^2+^_i_ in neurons exposed to AβO, probably due to the role of Src on NMDAR modulation [17]. Importantly, prior incubation of cells with antioxidants (GSH-EE; NAC; or Mitotempo, MT, a mitochondria-targeted superoxide dismutase mimetic) did not significantly change the Ca^2+^_i_ response in hippocampal neurons subjected to immediate AβO exposure (Figure 2C,D), suggesting that intraneuronal ROS generation and/or cellular oxidation events do not influence the increase in Ca^2+^_i_.

**Figure 3:**
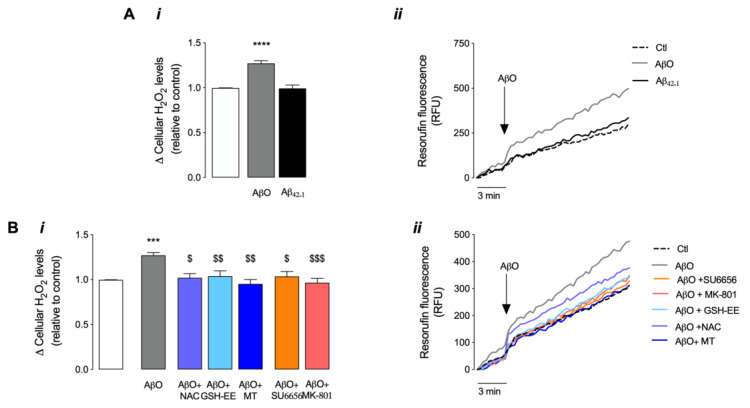
H_2_O_2_ levels following AβO stimulus in mature hippocampal neurons. Cellular H_2_O_2_ levels were evaluated by monitoring the fluorescence of resorufin. Basal fluorescence was recorded for 3 min, while the effect of AβO (1 μM) was recorded for 30 min (**A*ii***,**B*ii***). The effect of the reverse peptide Aβ_42–1_ (1 μM) was evaluated in (**A*i***,***ii***). The influence of NAC (1 mM), GSH-EE (0.1 mM) and Mitotempo (1 μM) or the effect of SU6656 (5 μM) and MK-801 (10 μM) were evaluated in (**B*i***,***ii***) in neurons exposed to AβO. In graphics (**i**), results were plotted as the difference between the last value achieved and the basal value before AβO addition, relative to the control. Graphics (**ii**) are the representative line charts (normalised to baseline). Data are expressed as the mean ± SEM of n = 3 to 10 experiments, run in quadruplicates. Statistical analysis: *** *p* < 0.001 or **** *p* < 0.0001 vs. control, ^$^ *p* < 0.05, ^$$^ *p* < 0.01 and ^$$$^ *p* < 0.001 vs. AβO (Kruskal-Wallis followed by Dunn’s post hoc test).

In Figure 3, we also evaluated the levels of H_2_O_2_ released by hippocampal neurons following immediate AβO (1 μM) exposure. Results evidence a significant rise in the levels of H_2_O_2_ released by cells right after AβO incubation; this result is specific to Aβ_1–42_ form, since the reverse peptide (Aβ_42–1_) did not induce significant changes in H_2_O_2_ levels (Figure 3A). Results depicted in Figure 3B demonstrate not only that pretreatment of hippocampal neurons with antioxidants (GSH-EE, NAC or MT) prevents the increase in H_2_O_2_ levels induced by the peptide oligomers but also that pre-incubation with MK-801 or SU6656 prevents the effect of AβO on H_2_O_2_ levels. These data suggest that, under these conditions, ROS generation is dependent on both NMDAR and Src activation.

Phosphorylation of the transcription factor Nrf2 (nuclear factor erythroid 2-related factor 2) at Ser40 is a signal for its nuclear translocation and an indicator of its activation following increased levels of cellular ROS [27]. Concordantly with AβO-immediate rise in H_2_O_2_ (data shown in Figures S1, S6 and S7) evidence a significant increase in the phosphorylation of Nrf2 at Ser40, starting as early as 5 min after AβO exposure, while extracellular H_2_O_2_ exposure was used as a positive control. 

These data show that AβO-induced Src activation is the result of a signaling pathway apparently initiated by the activation of NMDARs with the concomitant rise in Ca^2+^_i_, leading to enhanced H_2_O_2_ levels, which further evokes Src activation/phosphorylation. The observation of a time-dependent (5–30 min) decrease in P(Ser40)Nrf2/Nrf2 (Figures S1, S6 and S7) and an increase in P(Tyr416)Src/Src (Figure 1A) largely suggests that AβO-induced ROS generation linked to Nrf2 activation precedes Src activation in hippocampal neurons. Importantly, a feed-forward modulation of Src on both Ca^2+^_i_ and H_2_O_2_ levels induced by AβO cannot be excluded; data suggest that initial activation of Src after AβO exposure can evoke a continuous activation of NMDARs and a potential long-term amplification of AβO-induced effects in hippocampal neurons.

### 3.2. AβO-Induced Src Activation Regulates Mitochondrial H_2_O_2_ and Mild Mitochondrial Fragmentation

Aβ directly targets mitochondria [28,29], which also produce ROS, leading to the accumulation of ROS and subsequent oxidative unbalance. Of relevance, Src was found in mitochondria, where it can regulate cell survival by phosphorylating respiratory chain components [19], linking ROS signaling with mitochondrial function. Since AβO exposure leads to an increase in H_2_O_2_ in hippocampal neurons (Figure 3), we further investigated the impact of short exposure to AβO on mitochondrial H_2_O_2_ production and changes in mmp and the possible role of Src kinase (Figure S2).

**Figure 4:**
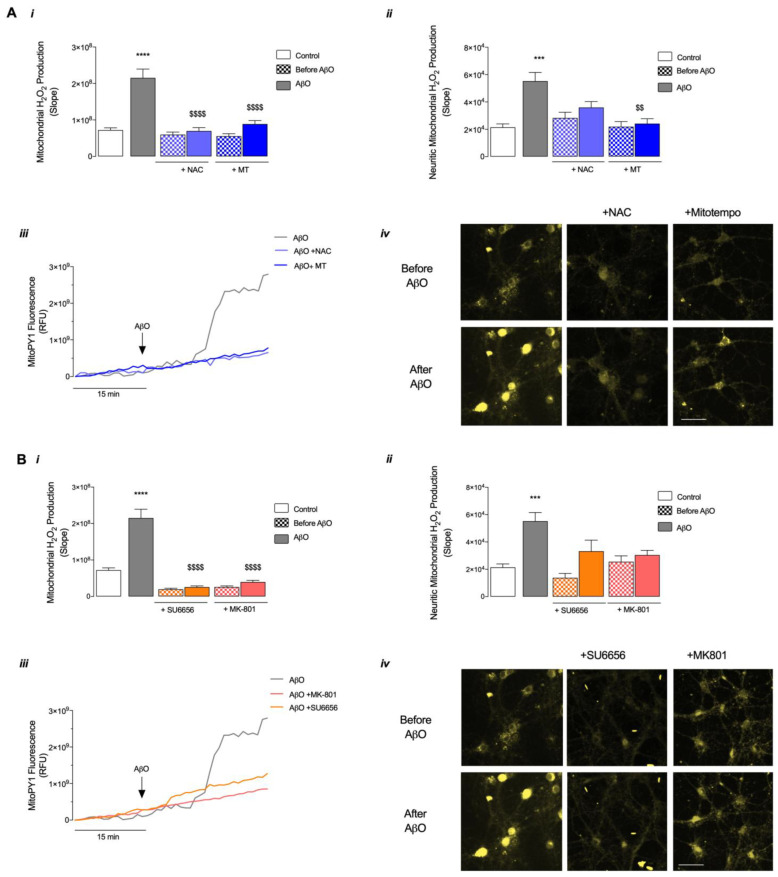
Mitochondrial H_2_O_2_ levels following AβO exposure in mature hippocampal neurons. (**A**,**B**) The levels of mitochondrial H_2_O_2_ were evaluated by monitoring the fluorescence of MitoPY1 (10 μM) in mature hippocampal neurons (17 DIV). Basal mitochondrial H_2_O_2_ was recorded for 15 min, and the effect of AβO (1 μM) was recorded for 30 min. The effects of Mitotempo (MT, 1 μM) and NAC (1 mM) (**A**) or SU6656 (5 μM) and MK-801 (10 μM) (**B**) were also evaluated. In graphics (***i***), the slope was calculated using values of RFU before and after AβO addition. Graphics (***ii***) slope was calculated by assessing fluorescence within neurites, only using values of RFU before and after AβO addition. (***iii***) Representative line charts (normalised to baseline). (***iv***) Fluorescence image of representative cells before and after the treatment. Scale bar: 50 μm. Data are the mean ± SEM of 20 to 100 single-cell analyses obtained from 2 to 5 independent experiments. Statistical analysis: *** *p* < 0.001 or **** *p* < 0.0001 vs. control/“no treatment” (Kruskal-Wallis followed by Dunn’s post hoc test); ^$$^ *p* < 0.01 or ^$$$$^ *p* < 0.0001 vs. AβO (Kruskal-Wallis followed by Dunn’s post hoc test).

Results depicted in Figure 4A,B show that 1 μM AβO induces a significant increase in the levels of mitochondrial H_2_O_2_. Interestingly, this increase occurs after a delay of about 15 min (Figure 4A*iii*,B*iii*), in contrast with the immediate changes in H_2_O_2_ cellular release observed in Figure 3, the latter probably resulting from an immediate AβO-evoked H_2_O_2_ production in the cytosol, prone to influence Nrf2 activation (Figure S1). As expected, both cellular and mitochondrial-selective antioxidants, namely NAC and MT, prevent the increase in overall mitochondrial H_2_O_2_ levels induced by the AβO stimulus (Figure 4A*i*,*iii*,*iv*). Furthermore, inhibition of NMDARs or Src also prevents AβO-induced mitochondrial H_2_O_2_ production (Figure 4B*i*,*iii*,*iv*); in this perspective, a direct effect of SU6656 on mitochondrial Src and its impact on mitochondrial H_2_O_2_ production cannot be excluded. Importantly, analysis of mitochondrial H_2_O_2_ specifically within neurites evidences similar results, although only MT is able to totally prevent the increase in mitochondrial H_2_O_2_ production induced by AβO stimulus (Figure 4A*ii*), while NAC (Figure 4A*ii*), MK-801 or SU6656 (Figure 4B*ii*) only partially prevent the effect mediated by the peptide oligomers. 

We further assessed if short exposure to AβO (1 μM) for 10 min (Figure S2A) or 30 min (Figure S2B) induces changes in mmp. Results shown in Figure S2 evidence no significant changes in mmp induced by short exposure to AβO, which is not influenced by MK-801 or SU6656 either.

Data indicate that mitochondrial H_2_O_2_ production is not an initial neuronal event after exposure to AβO, although it is largely influenced by NMDAR and Src activation and occurs without evidence of mitochondrial depolarization.

**Figure 5:**
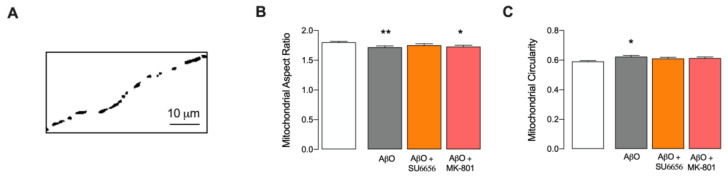
Dendritic mitochondrial morphology following AβO stimulus in mature hippocampal neurons. Cells cotransfected with pDsRed2-Mito and GFP plasmids were treated with AβO for 10 min, and mitochondrial morphology was measured using a 63× objective, NA = 1.4 on a spinning disk equipped Zeiss LSM 710 inverted microscope. The effect of SU6656 (5 μM) and MK-801 (10 μM) were also evaluated. (**A**) shows a representative mask of mitochondria obtained using the MitoProtAnalyser macro in Fiji used to assess mitochondrial morphology parameters, namely (**B**) aspect ratio and (**C**) circularity. Data are expressed as the mean ± SEM of n = 6–10 independent experiments, considering an average of 9 neuritis per analysis. Statistical analysis: * *p* < 0.05, ** *p* < 0.01 vs. Control (Kruskal-Wallis followed by Dunn’s post hoc test).

To deepen the impact of short AβO exposure and the role of Src on mitochondria, we further evaluated dynamic changes in mitochondrial morphology in mature hippocampal neurites (Figure 5). Figure 5A shows a representative mitochondrial mask obtained with the MitoProtAnalyser macro for Fiji that is further used by the macro to evaluate the mitochondrial aspect ratio (Figure 5B) and circularity (Figure 5C). Exposure to AβO for 10 min induces a slight but significant decrease in mitochondrial aspect ratio, which is prevented by pretreatment with SU6656 (Figure 5B). Importantly, the effect of AβO on mitochondrial aspect ratio occurs concomitantly with a slight but significant increase in mitochondrial circularity (Figure 5C), prevented both by SU6656 and MK-801. These results evidence that AβO short exposure induces mild mitochondrial fragmentation dependent on Src activation.

## 4. Discussion

AD is characterised by synaptic dysfunction and neuronal loss. The role of soluble species of Aβ in triggering neuronal dysfunction before cell death is largely accepted [30]. In previous studies, we demonstrated that AβO directly interact with NMDAR subunits (GluN1 and GluN2B) [20] evoking a transient increase in Ca^2+^_i_ [21]. AβO and agonist-selective NMDAR activation concur in increasing Ca^2+^_i_, which is rapidly and directly taken up by mitochondria and through the endoplasmic reticulum [6]. Excessive ROS production under these conditions [22] may then activate several redox-sensitive proteins, including Src kinase [14]. Indeed, our findings provide evidence that short exposure to AβO triggers the activation of Src through H_2_O_2_-dependent mechanisms, primarily involving the activation of NMDARs. This activation leads to an excessive buildup of Ca^2+^_i_, resulting in an imbalance of cytosolic and mitochondrial H_2_O_2_ levels. Interestingly, despite inducing mild mitochondrial fragmentation, brief AβO exposure per se does not cause significant persistent changes in mmp, a translation of its function.

Previous studies demonstrated that incubation of human and rat brain cortical cultures with aggregated Aβ_25–35_ induced a very rapid (about 1 min) and marked increase in Tyr phosphorylation of numerous neuronal proteins, including focal adhesion kinase (FAK). This effect was blocked by the addition of the SFK inhibitor 4-amino-5-(4-chlorophenyl)-7(t-butyl)pyrazol(3,4-d)pyramide (PP2) [31], suggesting that Aβ_25–35_ induced an almost immediate activation of SFKs. Conversely, intracerebroventricular injection of AβO in mice or 60 min incubation of hippocampal slices with AβO decreased Src phosphorylation [32]. Interestingly, we previously showed a decrease in Src phosphorylation/activation in the hippocampus and cortex of a 3-month-old 3xTg-AD mouse male, while an increase in Src phosphorylation was observed in the hippocampus of a 15-month-old 3xTg-AD mouse female [23], suggesting a dynamic regulation of this SFK. Herein, we observed a significant increase in the levels of phosphorylated Src after brief exposure (10, 30 min) to AβO, indicating that short exposure to AβO causes Src activation, while chronic exposure can be dynamically modulated so that a decrease in its activity might constitute a defense mechanism to avoid overstimulation of pathologically Src-dependent pathways. These differences may also be the result of the influence of other cell types; however, in primary hippocampal neurons cultured in the presence of a mitotic inhibitor, as ours, these are relatively absent and thus are not expected to contribute to modulate Src phosphorylation. 

In a model of chronic pain using inferior alveolar nerve transection (IANX), the authors observed the colocalization of Src and GluN1, an NMDAR subunit [33]. Interestingly, in this model, the administration of memantine (an antagonist of NMDAR) decreased IANX-induced upregulation of phosphorylated Src, while PP2 had no effect on GluN1 protein levels [33], suggesting a prior and required activation of NMDAR for Src activation. In an adult rat, the intracerebroventricular injection with Aβ_25–35_ induced Src-dependent Tyr phosphorylation of PSD-95 after the activation of GluN2A- and GluN2B-containing NMDARs [34]. In this study, we were able to decipher a sequence of cellular events, culminating in Src activation dependent on AβO-induced NMDAR activation, as demonstrated by the effect of MK-801. AβO-mediated initial NMDAR activation and Ca^2+^_i_ rise were associated with increased cytosolic H_2_O_2_ levels linked to Nrf2 phosphorylation/activation and a subsequent increase in mitochondrial H_2_O_2_ levels. Interestingly, in levodopa-induced dyskinesia, Src S-nitrosylation was caused by a neuronal nitric oxide synthase (nNOS)/NO signal activated by Ca^2+^ influx via GluN2B-containing NMDAR, which subsequently facilitated Src autophosphorylation (at Tyr416) and further phosphorylated GluN2B, demonstrating a positive feed-forward effect leading to GluN2B Tyr phosphorylation [35]. Our data is in agreement with a vicious cycle in which NMDAR-dependent Src activation/phosphorylation further promotes NMDAR activation.

Oxidative stress is one of the main processes involved in AD pathogenesis [36]. In former studies, Behl and colleagues demonstrated that Aβ (25–35 and 1–40 peptides) caused increased H_2_O_2_ levels and accumulation of lipid peroxides in primary cortical neurons, B12 and PC12 cells, affecting cell metabolism and survival [37]. Similar results were observed in Aβ-treated N2a neuroblastoma cells [38,39] and APP/PS1 transgenic cell lines associated with elevated Aβ levels, leading to cell death [38]. Herein, we evidence that acute AβO stimulus of mature hippocampal neurons is accompanied by an immediate increase in H_2_O_2_ released by cells and a delayed increase in mitochondrial H_2_O_2_ levels. Our data also show that the elevation of H_2_O_2_ levels is apparently the result of previous Ca^2+^_i_ dyshomeostasis, in accordance with previous findings [40]. Interestingly, we observe a primer role of non-mitochondrial/cytosolic H_2_O_2_ coincident with enhanced phosphorylation/activation of Nrf2 and subsequent P-Src and increased mitochondrial H_2_O_2_. In agreement, extracellular H_2_O_2_ targets mitochondria and induces increased mitochondrial ROS in SH-SY5Y human neuroblastoma cells [41]. Of note, Src was previously detected in mitochondria [18,42]. Moreover, we previously demonstrated that restoring active SFK levels improves mitochondrial morphology and function in a cellular model of Huntington’s disease [42]. These data suggest that changes in Src activation following short exposure to AβO might also occur within mitochondria. Since Src is involved in the modulation of brain mitochondrial respiration through the phosphorylation of complexes I, III and IV [19], changes in mitochondrial Src activation could reflect on mitochondrial function and thus potentiate the initial toxic effects of AβO. Nevertheless, no major changes in mmp were observed, which might be accounted for by a less sensitive fluorescence analysis of the overall cell population.

Several reports support mitochondrial dysfunction as an early event in AD etiology [43] and implicate Aβ in AD-associated mitochondrial dysfunction [44]. Apart from their important metabolic role, mitochondrial dynamic changes in morphology are also affected in AD. Exposure of Aβ to HT22 immortalised mouse primary hippocampal cells increased mitochondrial density and reduced mitochondrial length [45], suggesting mitochondrial fragmentation. Similarly, treatment of Wistar rats with Aβ_1–42_ induced a decrease in the levels of mitofusin-2 protein and translocation of Drp-1 to mitochondria [46], correlating with increased mitochondrial fragmentation. Primary hippocampal and cortical neurons from Tg2576 mice evidenced increased mitochondrial fission, decreased fusion and abnormal mitochondrial function [47]. Moreover, we previously demonstrated that prolonged AβO (1 μM, for up to 24 h) treatment caused mitochondrial fragmentation in mature primary hippocampal neurons [24] or in HT22 hippocampal cells without evidence of cell death [48]. Here, we evidence that short exposure to the same concentration of AβO is enough to induce a mild decrease in mitochondrial aspect ratio and an increase in mitochondrial circularity, supporting some degree of mitochondrial fragmentation. Importantly, these effects were completely prevented by the Src inhibitor SU6656 and only partially by the NMDARs antagonist MK-801, suggesting a possible role of specific mitochondrial Src on the modulation of AβO-induced mitochondrial morphology.

## 5. Conclusions

We demonstrate, for the first time, that brief exposure to AβO induces H_2_O_2_-dependent Src activation. Moreover, we evaluated potential sequential cellular events culminating in Src activation: (i) NMDARs activation linked to increased Ca^2+^_i_; (ii) cytosolic H_2_O_2_ levels unbalance and Nrf2 activation; and (iii) enhanced mitochondrial H_2_O_2_ levels and mild organelle fragmentation, associated with Src phosphorylation, anticipating its role in this organelle. 

In the past 10 years, interest in SFK targeting for the treatment of AD has increased. Fyn, an Src homolog, was the first to attract attention due to its interaction with both Aβ and Tau, as well as NMDARs [49]. This interest led to a phase Ib clinical trial (NCT01864655) to assess the safety, tolerability, and central nervous system availability of AZD0530 (saracatinib), an inhibitor of Src and Abl family kinases1 with a high potency for Src and Fyn in mild to moderate AD patients [50]. In the same year, AZD0530 was shown to rescue spatial memory deficits, synaptic depletion, Tau phosphorylation and deposition, and reduce microglial activation in APP/PS1 mice [51]. These were followed by a phase 2a randomised clinical trial (NCT02167256) to assess the effect of AZD0530 on cerebral metabolic decline in mild AD patients [52]. Unfortunately, there were no statistically significant effects of AZD0530 treatment on the cerebral metabolic rate for glucose, cognitive functions and total brain or ventricular volume, and patients exhibited several adverse events; however, it showed a trend for slowing down the reduction in hippocampal volume and entorhinal thickness [52]. Based on these results and our data, novel compounds ought to be produced to improve their selectivity towards specific SFK members, which could be tested in earlier stages of AD aiming to prevent major changes in mitochondria and hippocampal glutamatergic synapses.

## Data Availability

Data and materials described in the present manuscript will be available upon adequate request.

## Supplementary Materials

The following supporting information can be downloaded at https://www.mdpi.com/article/10.3390/antiox12091770/s1, Figure S1: Nrf2 total and phosphorylated protein levels in mature hippocampal neurons after AβO exposure; Figure S2: Mitochondrial membrane potential following AβO exposure in mature hippocampal neurons; Figure S3: Entire Western blot membranes which correspond to the cropped Western blot band shown in Figure 1Aii and 1A*iii*; Figure S4: Entire Western blot membranes which correspond to the cropped Western blot band shown in Figure 1B*ii* and 1B*iii*; Figure S5: Entire Western blot membranes which correspond to the cropped Western blot band shown in Figure 1C*ii* and 1C*iii*; Figure S6: Entire Western blot membranes which correspond to the cropped Western blot band shown in Figure S1A; Figure S7: Entire Western blot membranes which correspond to the cropped Western blot band shown in Figure S1B.
