# Supplementary material for: Uncovering the Early Events Associated with Oligomeric Aβ-Induced Src Activation
Source: Antioxidants (Basel). 2023 Sep 16;12(9):1770. doi: 10.3390/antiox12091770 (PMC10525724; doi:10.3390/antiox12091770)
Supplement: Supplementary file 1 [file antioxidants-12-01770-s001.zip › antioxidants-2600019-supplementary.pdf]

Supplementary Data

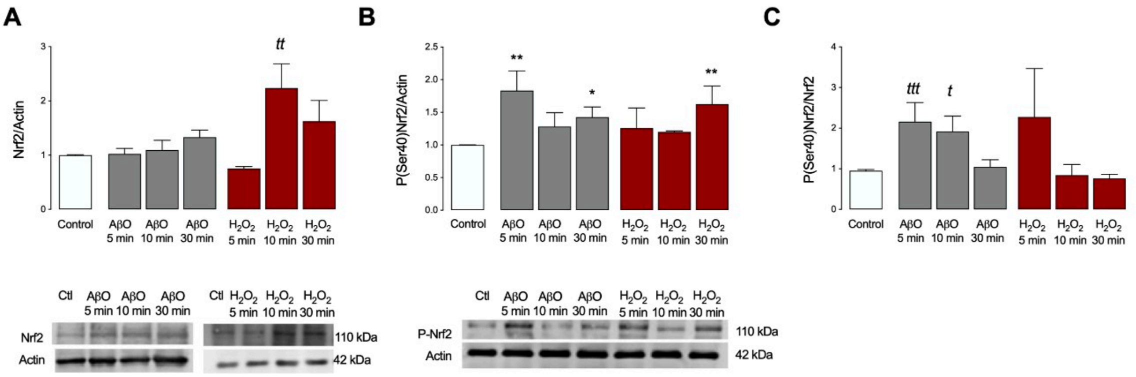

Figure S1. Nrf2 total and phosphorylated protein levels in mature hippocampal neurons after AβO exposure. Hippocampal mature neurons were incubated with 1 μM AβO for 5, 10 and 30 min and the levels of Nrf2/actin (A), P(Ser40)Nrf2/actin (B) and P(Ser40)Nrf2/Nrf2 (C) in total cell extract were evaluated using Western blotting. Data are expressed in arbitrary units relative to actin as the mean ± SEM of n=3 to 11 experiments. Statistical analysis: *$p < 0.05$ or **$p<0.01$ vs. Control (Kruskal-Wallis followed by Dunn's post hoc test); $^{t}p < 0.05$, $^{tt}p < 0.01$ and $^{ttt}p < 0.001$ vs. Control (Mann-Whitney).

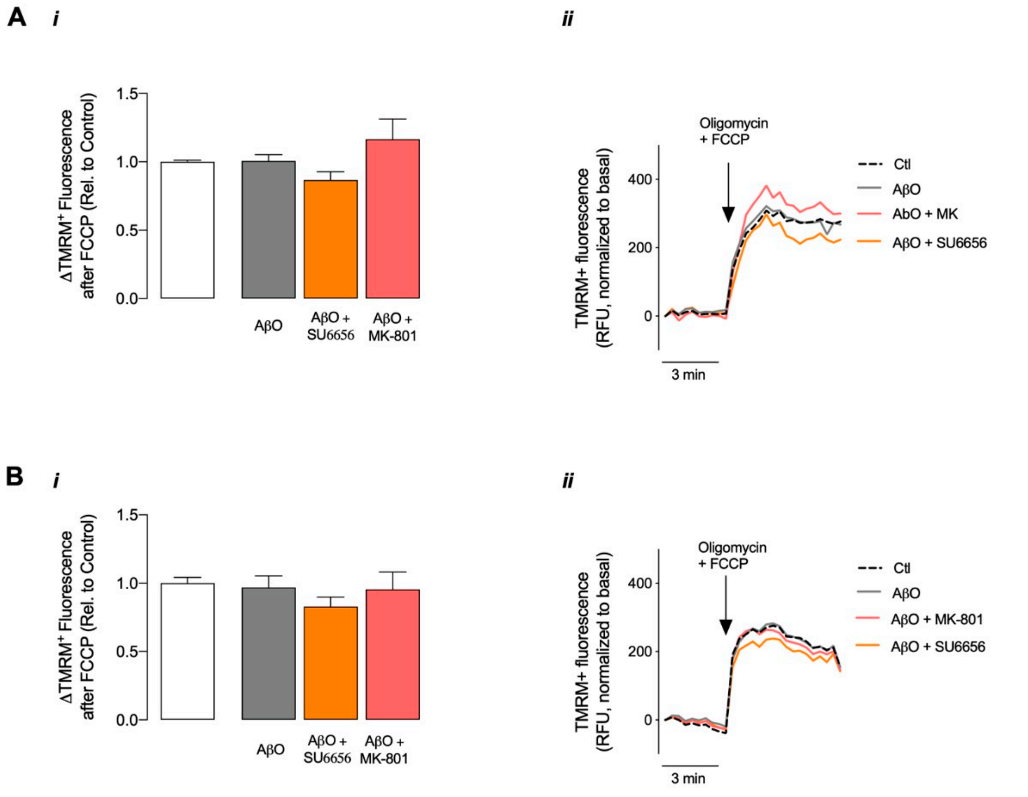

Figure S2. Mitochondrial membrane potential following AβO exposure in mature hippocampal neurons. AβO-induced changes in mitochondrial membrane potential were determined using $TMRM^+$ under quenched conditions. Cells were incubated with AβO (1 μM) for 10 min (A) or 30 min (B). The effect of SU6656 (5 μM) and MK-801 (10 μM) were evaluated (A-B). Mitochondrial membrane potential was assessed after complete mitochondrial depolarisation using oligomycin plus FCCP (2 μg/ml and 2 μM, respectively). In graphics (i), results were plotted as the difference between the maximal value achieved and the basal value before AβO addition, relative to control. Graphics (ii) are the representative line charts. Data are expressed as the mean ± SEM of n=3 to 9 experiments, run intriplicate.

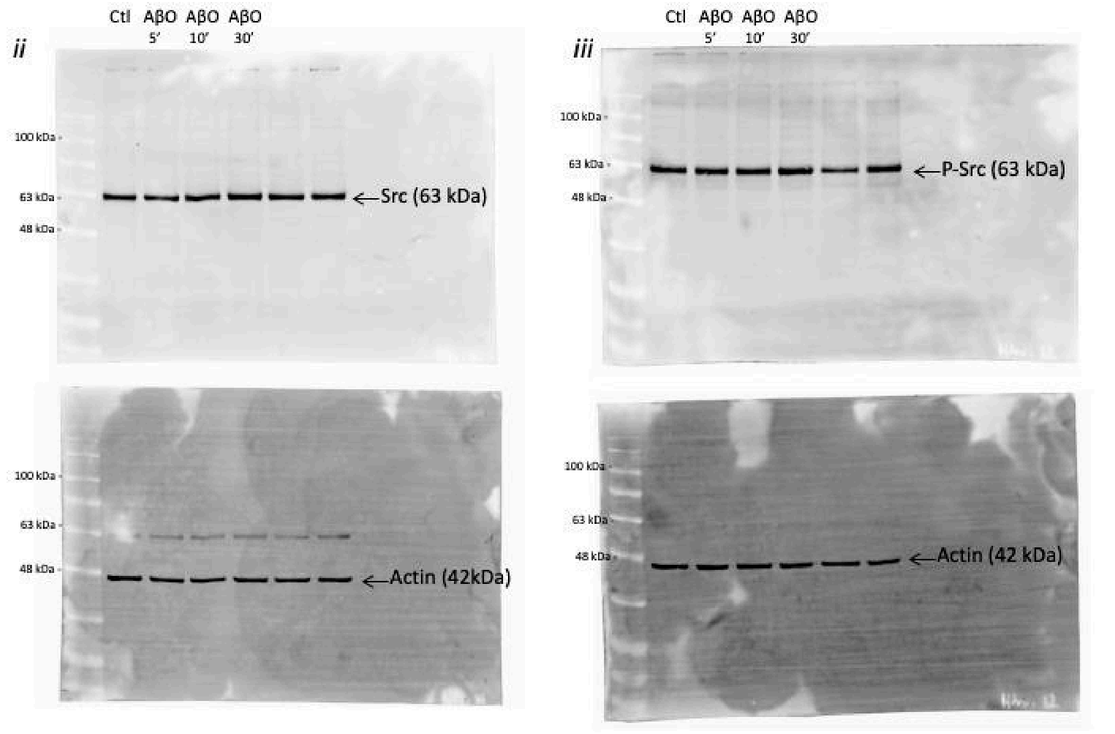

Figure S3: Entire Western blot membranes, which correspond to the cropped Western blot band shown in Figure 1A*ii* and 1A*iii*.

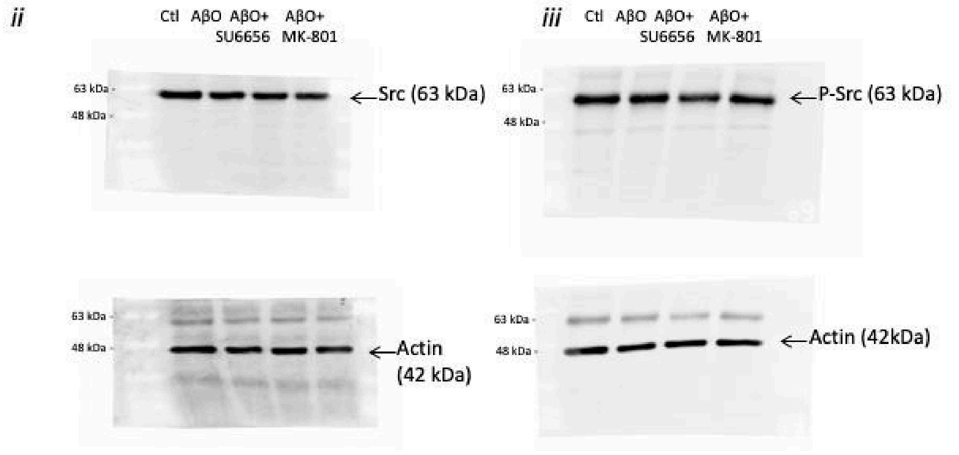

Figure S4: Entire Western blot membranes, which correspond to the cropped Western blot band shown in Figure 1B*ii* and 1B*iii*. Note that the membranes have been cut before incubation with the antibodies.

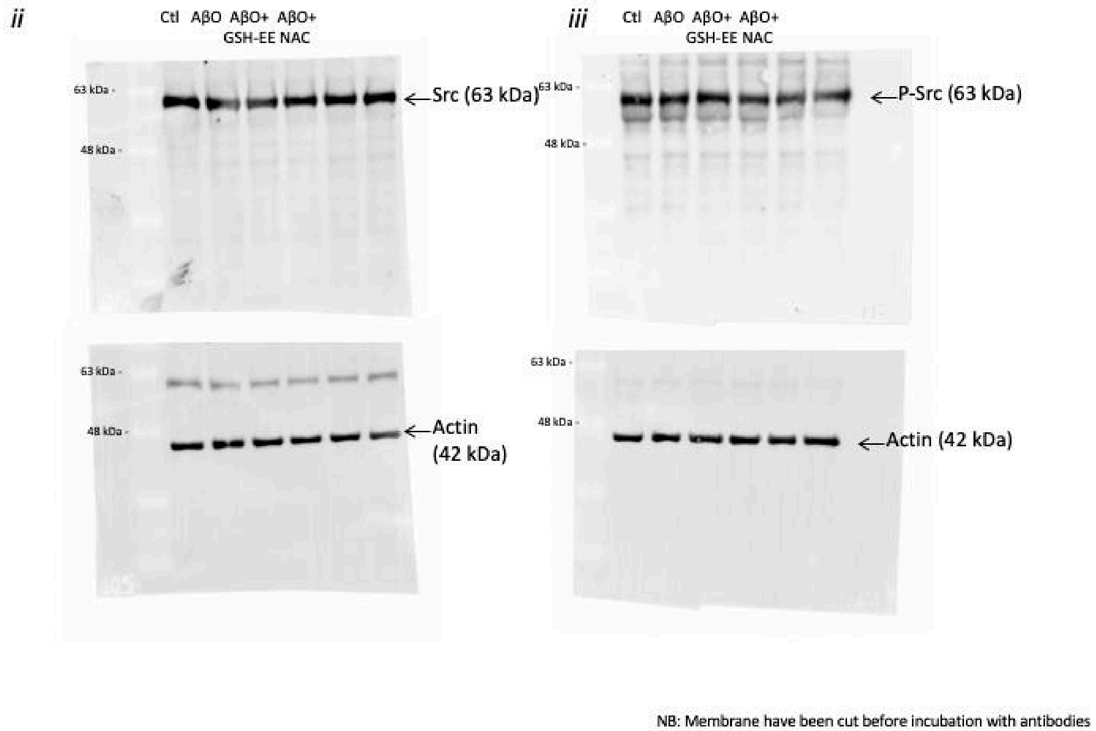

Figure S5: Entire Western blot membranes, which correspond to the cropped Western blot band shown in Figure 1C*ii* and 1C*iii*. Note that the membranes have been cut before incubation with the antibodies.

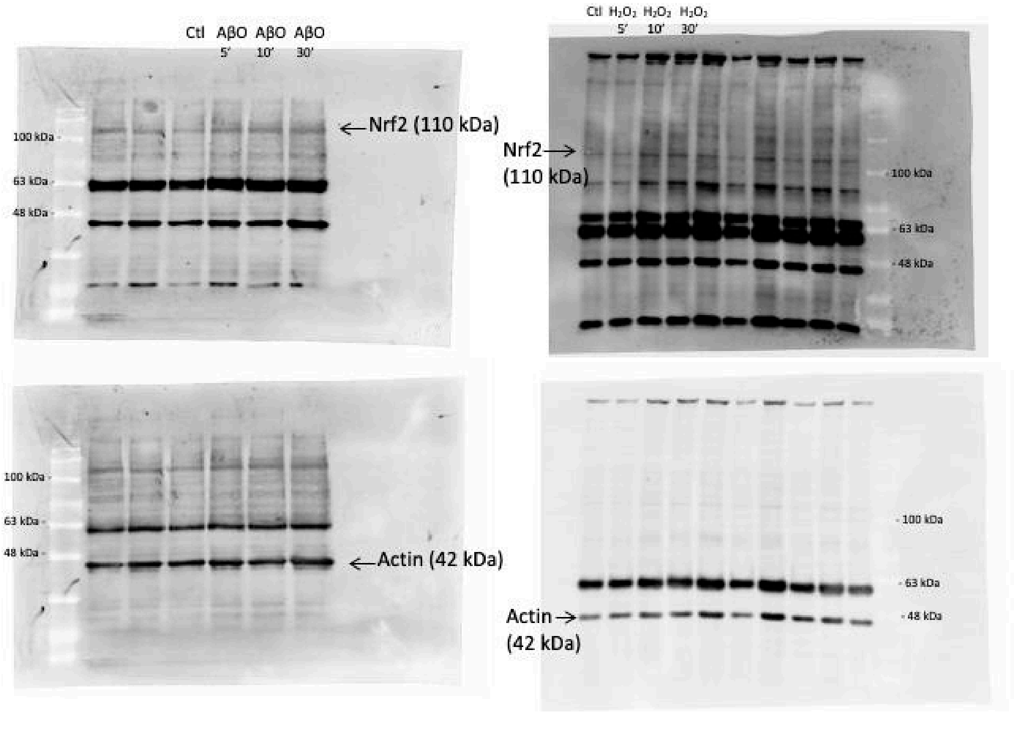

Figure S6: Entire Western blot membranes, which correspond to the cropped Western blot band shown in Figure S1A.

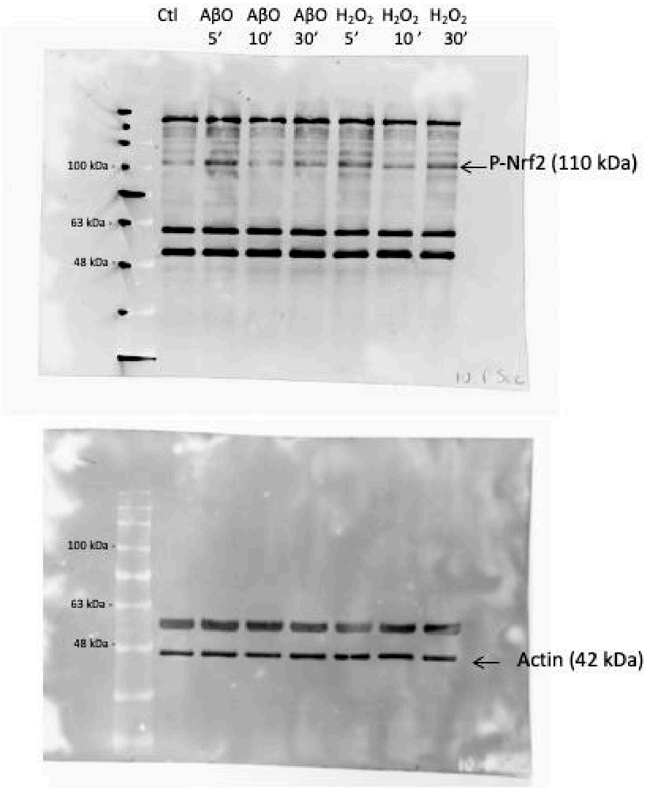

Figure S7: Entire Western blot membranes, which correspond to the cropped Western blot band shown in Figure S1B.
